# Comparison of Policosanols via Incorporation into Reconstituted High-Density Lipoproteins: Cuban Policosanol (Raydel^®^) Exerts the Highest Antioxidant, Anti-Glycation, and Anti-Inflammatory Activity

**DOI:** 10.3390/molecules28186715

**Published:** 2023-09-20

**Authors:** Kyung-Hyun Cho, Ji-Eun Kim, Hyo-Seon Nam, Dae-Jin Kang, Seung-Hee Baek

**Affiliations:** 1 Raydel Research Institute, Medical Innovation Complex, Daegu 41061, Republic of Korea; ths01035@raydel.co.kr (J.-E.K.); sun91120@raydel.co.kr (H.-S.N.); daejin@raydel.co.kr (D.-J.K.); shbaek@raydel.co.kr (S.-H.B.); 2 LipoLab, Yeungnam University, Gyeongsan 38541, Republic of Korea

**Keywords:** HDL, high-density lipoproteins, apolipoprotein A-I, policosanol, sugar cane wax alcohol, zebrafish, embryo

## Abstract

Reconstituted high-density lipoproteins (rHDL) containing each policosanol from Cuba (Raydel^®^), China (Shaanxi Pioneer), and the United States (Lesstanol^®^) were synthesized to compare the physiological properties of policosanol depending on sources and origin countries. After synthesis with apolipoproteinA-I (apoA-I) into rHDL, all policosanols bound well with phospholipid and apoA-I to form discoidal rHDL. An rHDL containing Cuban policosanol (rHDL-1) showed the largest rHDL particle size of around 83 ± 3 nm, while rHDL containing Chinese policosanol (rHDL-2) or American policosanol (rHDL-3) showed smaller particles around 63 ± 3 nm and 60 ± 2 nm in diameter, respectively. The rHDL-1 showed the strongest anti-glycation activity to protect the apoA-I degradation of HDL from fructose-mediated glycation: approximately 2.7-times higher ability to suppress glycation and 1.4-times higher protection ability of apoA-I than that of rHDL-2 and rHDL-3. The rHDL-1 showed the highest antioxidant ability to inhibit cupric ion-mediated LDL oxidation in electromobility and the quantification of oxidized species. A microinjection of each rHDL into a zebrafish embryo in the presence of carboxymethyllysine (CML) showed that rHDL-1 displayed the strongest anti-oxidant activity with the highest embryo survivability, whereas rHDL-2 and rHDL-3 showed much weaker protection ability, similar to rHDL alone (rHDL-0). An intraperitoneal injection of CML (250 μg) into adult zebrafish caused acute death and hyperinflammation with an elevation of infiltration of neutrophils and IL-6 production in the liver. On the other hand, a co-injection of rHDL-1 resulted in the highest survivability and the strongest anti-inflammatory ability to suppress IL-6 production with an improvement of the blood lipid profile, such as elevation of HDL-C and lowering of the total cholesterol, LDL-cholesterol, and triglyceride. In conclusion, Cuban policosanol exhibited the most desirable properties for the in vitro synthesis of rHDL with the stabilization of apoA-I, the largest particle size, anti-glycation against fructation, and antioxidant activities to prevent LDL oxidation. Cuban policosanol in rHDL also exhibited the strongest in vivo antioxidant and anti-inflammatory activities with the highest survivability in zebrafish embryos and adults via the prevention of hyperinflammation in the presence of CML.

## 1. Introduction

Several studies have shown that higher serum high-density lipoproteins-cholesterol (HDL-C) level is associated with healthy longevity [1,2], while the serum levels of total cholesterol (TC) and low-density lipoproteins cholesterol (LDL-C) are not associated with longevity. In addition to the HDL quantity, increasing the HDL functionality is also essential to maintain a healthy life to suppress the incidence of aging-related diseases, such as hypertension, metabolic syndrome, rheumatoid arthritis, and dementia [3].

Many pharmaceuticals and nutraceuticals, such as niacin and CETP inhibitors, have been developed to raise the HDL-C level [4]. On the other hand, they have mostly failed due to the off-target effect and severe side effects, such as increased blood pressure [5], accumulation in adipose tissue [6], and face flushing [7]. Although a few synthetic CETP inhibitors could raise the HDL-C quantity significantly, it was not confirmed whether the HDL particle quality and functionality were also improved by CETP inhibition.

In order to treat dyslipidemia, policosanols have been used as a nutraceutical and pharmaceutical ingredients, which were purified from many plant and insect sources, with a mixture of aliphatic alcohols ranging from 24 to 34 carbon atoms, such as octacosanol, triacontanol, dotriacontanol, hexacosanol, and tetratriacontanol as the major components. Among them, Cuban sugarcane (*Saccharum officinarum* L.) wax alcohol, policosanol (PCO), was reported to have a beneficial effect in increasing the HDL-C quantity and enhancing HDL quality and functionality after incorporation into reconstituted HDL with potent CETP inhibition activity [8]. The improvement of the HDL-C quantity and quality was linked with the blood pressure-lowering effect in spontaneous hypertensive rats, prehypertensive Korean participants, and middle-aged Japanese participants. Moreover, 12 weeks of consumption of Cuban policosanol (Raydel^®^) in the middle-aged participants offered protection of liver functions and kidney functions with a decrease in glycated hemoglobin and blood pressure [9].

Since the first report on policosanol in Cuba was published in 1993 [10], with a genuine composition of 1-tetracosanol (C_24_H_49_OH, 0.1–20 mg/g); 1-hexacosanol (C_26_H_53_OH, 30.0–100.0 mg/g); 1-heptacosanol (C_27_H_55_OH, 1.0–30.0 mg/g); 1-octacosanol (C_28_H_57_OH, 600.0–700.0 mg/g); 1-nonacosanol (C_29_H_59_OH, 1.0–20.0 mg/g); 1-triacontanol (C_30_H_61_OH, 100.0–150.0); 1-dotriacontanol (C_32_H_65_OH, 50.0–100.0 mg/g); 1-tetratriacontanol (C_34_H_69_OH, 1.0–50.0 mg/g) [11], many other brand names of policosanols have been developed from different sources and origins of countries for global marketing. Policosanols can be purified from many waxes or various plants, such as oats [12], insects [13], and beeswax [14]. Many studies have reported the multi-aspect of the various policosanols from different sources. In particular, rice bran policosanol also showed an antidiabetic effect [15] and attenuation of the thrombosis effect [16]. On the other hand, despite the variety of policosanol, except in a preceding paper [17], no study has directly compared the policosanols from different origins of sources and brands regarding the correlations between the chemical compositions and physiological effects, such as high-density lipoproteins (HDL) binding ability and enhancement of HDL functionalities.

Although many products of policosanols, at least 11 different kinds of brand names, have been marketed worldwide, the efficacy still needs to be directly compared despite their different ingredient compositions. Furthermore, some copycat policosanol products showed unidentified ingredient compositions and no proof of efficacy and safety data, contributing to the impaired reputation of genuine policosanol. In this context, it is a comparison study of policosanol compositions and efficacies regarding in vitro characterization in rHDL and in vivo efficacy to treat hyperinflammation. These in vitro and in vivo evaluations of various policosanols in the rHDL state can provide useful information for better consumer choices.

The current study compared the policosanols from Cuba (sugarcane, PCO1), China (rice bran, PCO2), and the USA (sugarcane, PCO3) in terms of physicochemical characterizations, such as the particle shape and size, after encapsulating each policosanol in rHDL. The individual rHDL comprising different policosanol (rHDL-PCO) was examined for their structure and functionality with respect to antioxidant, anti-glycation, and anti-inflammatory activities using in vitro and in vivo studies employing embryos and adult zebrafish. The anti-glycation activity of rHDL comprising various PCO was examined as an earlier described method [8] using fructose, which is reported as a potent glycation agent compared to glucose [18] and leads to the effective formation of advanced glycation end products (AGEs). Amidst AGEs, *N*-ε-carboxymethyllysine (CML) abundance in serum is often associated with modifying lipoproteins and provides a suitable environment for LDL oxidation, thus significantly contributing to atherosclerosis [19]. Also, higher serum CML interacts with highly sensitive C-reactive proteins (CRP), provoking TLR expression in monocytes leading to a pro-inflammatory state [20]. The comparative protective effects of various PCOs against CML-posed toxicity were accessed in zebrafish embryos by monitoring development deformities, swimming activity, and mortality after injecting rHDL-PCO.

Zebrafish (*Danio rerio*) are an excellent vertebrate model for inflammation and metabolic disorders due to their higher genetic similarity with humans. Zebrafish have a well-developed immune system that responds similarly to the mammalian immune system [21]. Moreover, zebrafish harbor transparent embryos that develop externally and respond efficiently against different stimuli; therefore, they are utilized in various medicinal studies, including assessing the teratogenic effects and inflammation of drug candidates [22]. The anti-inflammatory properties of various policosanols were compared using zebrafish embryos by testing the developmental speed, morphology, and survivability after injecting rHDL containing each policosanol. An intraperitoneal injection of CML, 250 μg and 500 μg, into normolipidemic and hyperlipidemic zebrafish, respectively, as an in vivo test, caused acute paralysis and death with a loss of swimming ability [23].

The current study aims to compare the physiological characteristics of the different policosanols after incorporation with rHDL. Further, the functionality of each rHDL-PCO was assessed in mitigating glycation induced by fructose in HDL, as well as its antioxidant potential in terms of suppressing the production of reactive oxygen species (ROS) induced by CML and averting apoptosis in zebrafish embryos. Finally, the effect of rHDL-PCO was evaluated against CML-induced acute hepatotoxicity and dyslipidemia in adult zebrafish.

## 2. Results

### 2.1. Ingredient Composition Analysis

The three policosanols showed distinctly different amounts of wax alcohols and compositions of long-chain aliphatic alcohols (Table 1). Cuban policosanol (PCO1) showed the highest total amount of wax alcohol (~982 mg/g) and octacosanol content (692 mg, 70.5%). In contrast, Chinese policosanol (PCO2) had the lowest total amount of wax alcohol (~739 mg/g) and the lowest octacosanol content (~56 mg, 7.6%). American policosanol (PCO3) had the 2nd highest amount of total wax alcohol (~900 mg/g) and octacosanol (546 mg, 60.7%), suggesting similar C27, C30, and C32 contents to Cuban policosanol. Hence, the three policosanols had different ingredient compositions, but the American policosanol (from sugar cane wax) had more similarity to Cuban policosanol (from sugar cane wax) than Chinese policosanol (from rice bran). The powder image of Cuban policosanol revealed a more beige color than other policosanols with different textures (Table 1).

### 2.2. Synthesis of rHDL with Policosanol

**Figure 1:**
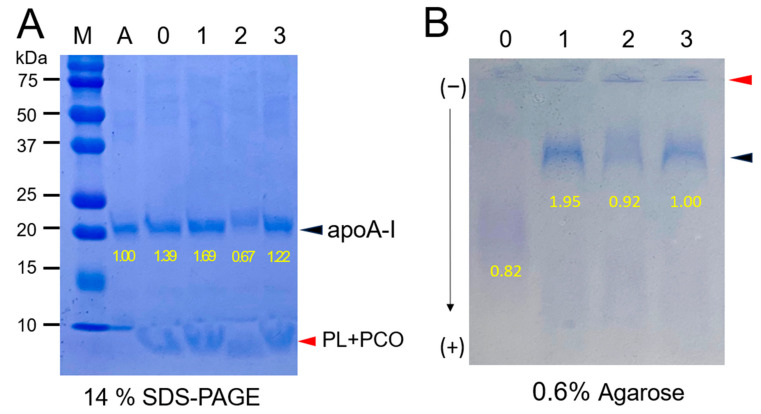
Electrophoresis of reconstituted high-density lipoproteins (rHDL) containing each policosanol (PCO) after synthesis with palmitoyloleoylphosphatidylcholine (POPC), free cholesterol (FC), and apolipoprotein A-I (apoA-I). The molar ratio of POPC:FC:apoA-I:PCO = 95:5:1:1. Yellow font numbers indicate band intensity in each lane. (**A**) Electrophoretic mobility of denatured rHDL (5 μg/well) in 14% SDS-PAGE. A red arrow indicates PCO and phospholipid (PL) debris. Lane M and lane A contain the standard protein molecular weight marker (Bio-Rad 161-0374) and rHDL devoid of PCO, respectively, while lanes 1, 2, and 3 comprise rHDL with PCO1, PCO2, and PCO3, respectively. The gel was stained with Coomassie brilliant blue (final, 0.125%) to visualize separated protein (apo A-I) and phospholipids (PL). (**B**) Comparative electrophoretic mobility of rHDL (15 μg/well) in the native state employing 0.6% agarose gel. Coomassie brilliant blue (final, 1.25%) was used to visualize apo A-I in rHDL. Red and black arrowhead indicates loading position and rHDL band containing each policosanol, respectively.

The three policosanols exhibited sufficient binding ability with phospholipid and apoA-I, as shown in Figure 1A, even though PCO2 showed a faint band intensity of apoA-I with a notable upshift band position, as indicated in the black arrowhead. The phospholipid and policosanol mixture in each rHDL was detected in the bottom of the gel (14% SDS-PAGE), as indicated by the red arrowhead. The electromobility of PCO2 (lane 2, Figure 1A) was faster than that of PCO1 and PCO3. As shown in Figure 1B, in agarose gel electrophoresis, the three reconstituted HDL-containing policosanols showed similar electromobility, which was slower than rHDL without policosanol (rHDL-0). Interestingly, rHDL-1 (lane 1) showed the most distinct band intensity and the least aggregation in the loading position, as indicated by the red arrowhead. In contrast, rHDL-2 and rHDL-3 (lanes 2 and 3, respectively) showed weaker band intensity and were more aggregated in the loading position. In particular, rHDL-2 showed the weakest band intensity in agarose gel (lane 2, Figure 1B), indicating the putative proteolytic degradation or modification of apoA-I by PCO2 as observed in SDS-PAGE (lane 2, Figure 1A). These results suggest that rice bran policosanol (PCO2) might have the different binding abilities with apoA-I and phospholipid to alter the electromobility from sugarcane wax policosanol (PCO1 and PCO3).

### 2.3. Reconstituted HDL Particle Analysis

Wavelength maximum fluorescence (WMF) analysis revealed that reconstituted HDL without policosanol (rHDL-0) had 329.2 nm of WMF (Table 2), while lipid-free apoA-I showed 334.3 nm, indicating a 5.1 nm blue shift of WMF in apoA-I. This result suggests that intrinsic Trp residue in apoA-I moved to a more nonpolar phase upon binding with phospholipid and cholesterol. An rHDL containing PCO-1 (rHDL-1) showed approximately 325.2 nm of WMF, a 4.0 nm larger blue shift of WMF in apoA-I, suggesting that PCO1 binding caused more movement of intrinsic Trp toward the nonpolar phase. On the other hand, an rHDL containing PCO-2 (rHDL-2) and rHDL containing PCO-3 (rHDL-3) showed a smaller blue-shift of WMF of approximately 2.7 nm and 1.9 nm, respectively, suggesting less movement of Trp toward the nonpolar phase.

**Figure 2:**
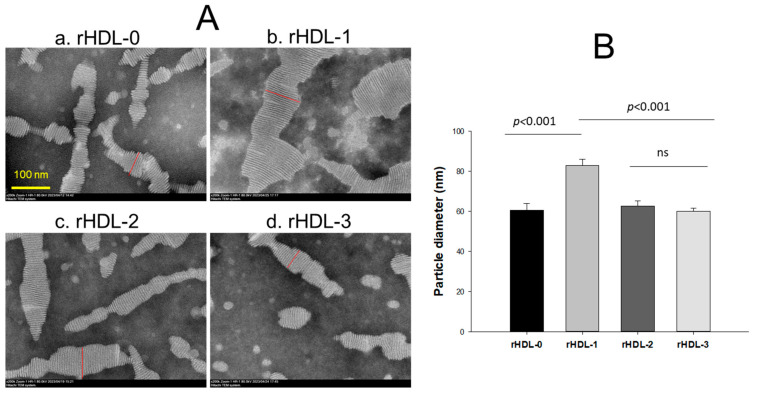
Transmission electron microscopy (TEM) image of each reconstituted high-density lipoproteins (rHDL)-containing policosanol and measurement of the particle diameter. (**A**) Comparison of the particle morphology with 40,000× magnification. All rHDL showed a discoidal shape and a rouleaux pattern. Red line indicates direction of particle diameter. (**B**) Measurement of the mean particle diameter among rHDL.

Transmission electron microscopy (TEM) revealed the rHDL-1 to have the most distinct disc particle shape with a rouleaux morphology and the highest particle number with the largest particle size (Figure 2A). The particle size of the rHDL-1 was 1.6-fold and 1.3-fold larger than rHDL-0 and rHDL-2, respectively (Figure 2B). Although all rHDL-containing policosanols were larger than rHDL-0, rHDL-3 showed the smallest particle number and a severely unclear and ambiguous morphology, approximately 60 nm in diameter. These results suggest that different origins of policosanol might have different abilities to form rHDL upon different binding affinities with phospholipid and apoA-I.

### 2.4. Anti-Glycation Activity of rHDL

Fructated (final 250 mM of fructose) human HDL (2 mg/mL of total protein) exhibited severe glycation with a 6.9-fold higher yellowish fluorescence intensity (FI) than HDL alone during 72 h incubation under 5% CO_2_. Contrarily, co-treatment of rHDL-1 caused the remarkable inhibition of glycation of HDL up to 29% lower FI extent than HDL alone, while the other rHDL showed no significant inhibition, approximately 9–19% inhibition during the 72 h incubation.

Interestingly, rHDL-0 showed higher inhibition ability (~19% inhibition) than rHDL-2 (~9% inhibition) and rHDL-3 (~13% inhibition) against fructation, suggesting that the incorporation of PCO2 and PCO3 in rHDL had little effect on preventing glycation or could not facilitate of the anti-glycation activity of rHDL.

**Figure 3:**
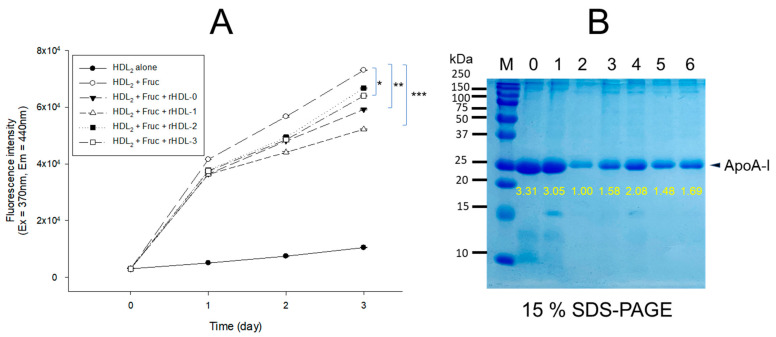
Anti-glycation activity of reconstituted high-density lipoproteins (rHDL)-containing policosanol in glycated HDL through fructose (Fruc, final 250 mM) treatment under 5% CO_2_ at 37 °C. (**A**) Fluorescence spectroscopic analysis (Ex = 370 nm, Em = 440 nm) of HDL (2 mg/mL of protein), which was co-treated with fructose (final 250 mM) and each rHDL (2 mg/mL of apoA-I) containing policosanol (final 3 μg/mL) during 72 h incubation. The data are expressed as the mean ± SD from three independent experiments with duplicate samples. * *p* < 0.05 vs. HDL + Fruc; ** *p* < 0.01 vs. HDL + Fruc; *** *p* < 0.001 vs. HDL + Fruc. Each rHDL treatment was compared with HDL + Fruc by paired *t*-test. (**B**) Electrophoretic patterns of the HDL (5 μg/lane) after incubation with fructose and each rHDL after 72 h incubation (15% SDS-PAGE). Yellow font numbers indicate band intensity in each lane. Lane 0, HDL alone at 0 h incubation; lane 1, HDL alone at 72 h incubation; lane 2, HDL + Fruc; lane 3, HDL + Fruc + rHDL-0; lane 4, HDL + Fruc + rHDL-1; lane 5, HDL + Fruc + rHDL-2; lane 6, HDL + Fruc + rHDL-3.

As shown in Figure 3B, electrophoresis with each HDL_2_ sample exhibited that HDL_2_ alone had a distinct apoA-I band (28 kDa, lane 1) after seven days of incubation. In contrast, the fructated HDL_2_ (lane 2) showed a remarkably decreased apoA-I band with proteolytic degradation and aggregation. On the contrary, rHDL-1-treated HDL_2_ showed the strongest apoA-I band (lane 4) without protein aggregation: two-fold higher band intensity than HDL_2_ + fructose. Interestingly, the rHDL-0 treatment (lane 3) exhibited more protection of the apoA-I band than that of rHDL-2 (lane 5) and rHDL-3 (lane 6), indicating that the apoA-I band of the other rHDL treatment disappeared with severe protein aggregation. Hence, Cuban policosanol (PCO1) in rHDL-1 could inhibit the glycation of HDL_2_ and protect apoA-I from degradation in the presence of high fructose concentrations (final 250 mM). In contrast, Chinese (PCO2) and American (PCO3) policosanol did not exhibit significant anti-glycation activity.

### 2.5. Anti-Oxidant Activity against LDL Oxidation

**Figure 4:**
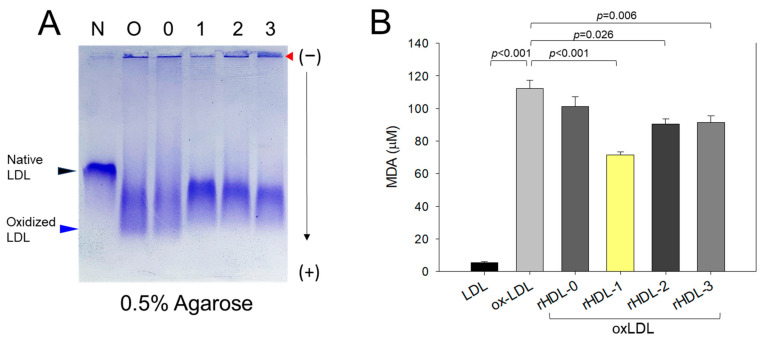
Comparative antioxidant potential of reconstituted high-density lipoproteins (rHDL)-comprising policosanol to prevent cupric ion mediated oxidative damage of LDL. (**A**) Agarose gel electrophoresis of LDL (10 μg of protein) treated with rHDL (0.5 μg of protein) comprising different origin of policosanol. The native LDL and oxLDL were separated in 0.5% agarose gel using Tris-EDTA buffer (pH 8.0) at 50 V for 1 h. The separated bands of the apo-B fraction of LDL were stained with Coomassie brilliant blue (final 1.25%). (**B**) Quantification of thiobarbituric acid reactive substances (TBARS) in LDL challenged with cupric ion and subsequently treated with rHDL comprising policosanol. The values were represented as malondialdehyde (MDA) (μM) in LDL using the MDA standard. Values in the bar graph represent the mean ± SD of three independent experiments. A pairwise statistical difference was established using *t*-test by comparing the results with the ox-LDL value. Red arrow indicates loading position of each well.

In the native state, LDL showed retarded electrophoretic mobility with an intact sharp band without any smearing and aggregation in the loading position (indicated by a black arrow in Figure 4A). Disparate to this CuSO_4_ (final 10 μM)-treated LDL (oxLDL) showed faster electrophoretic mobility with faint band intensity and smearing (as indicated by the blue arrow) owing to the oxidative damage of the apo-B in the oxLDL. Also, the aggregation of LDL in the loading well was observed, signifying the oxidative damage of LDL (lane O, Figure 4A). However, the rHDL-1 treatment efficiently prevent LDL oxidation induced by cupric ions, as evident by a sharp intact band with retarded electrophoretic mobility and least accumulation of LDL in the loading well position (lane 1, Figure 4A). Contrary to this, LDL + CuSO_4_ treated with rHDL-2 or rHDL-3 showed slightly higher electrophoretic mobility with the appearance of diffused bands and high protein accumulation in the respective loading wells, suggesting less effect on the prevention of LDL oxidation. Precisely, LDL treated with rHDL-3 (lane 3) displayed the least preventive effect against Cu^2+^-posed oxidative damage of LDL, and this effect resembles the impact exerted by rHDL-0 (lane 0, Figure 4A). These results indicate that the rHDL1 displayed the highest antioxidant activity that effectively blocked the cupric ion mediated oxidative damage of LDL.

Furthermore, the degree of oxidative damage of LDL and the preventive role of rHDLs were examined by TBARS assay (Figure 4B). The findings suggested a significantly 23-fold higher malondialdehyde (MDA) content in LDL treated with CuOS_4_ compared to native LDL. In contrast, a 38% lower MDA level was quantified in LDL treated with rHDL-1, signifying Cuban policosanol’s impact in inhibiting Cu^2+^-mediated oxidative damage. Contrary to this, the rHDL-2 and rHDL-3-treated LDL showed a significant 21% and 19% reduced MDA level than that of oxLDL control, attesting to their impact on the inhibition of LDL oxidation; however, the impact was much lower than that of rHDL-1. These results suggest that PCO1 (Cuban policosanol) exerts remarkably higher antioxidant ability against cupric ion-mediated LDL oxidation than PCO2 (Chinese policosanol) and PCO3 (American policosanol).

### 2.6. Antioxidant Activity against CML Toxicity in Embryo

**Figure 5:**
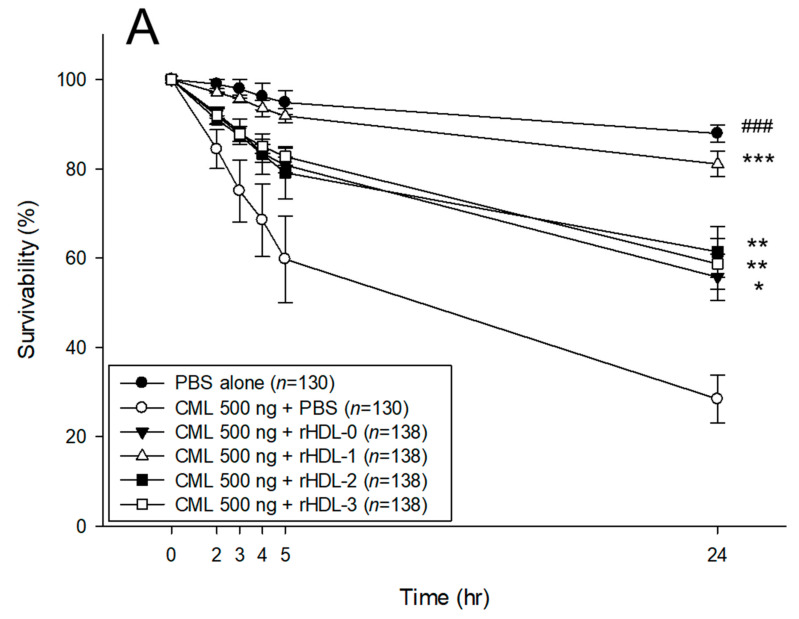

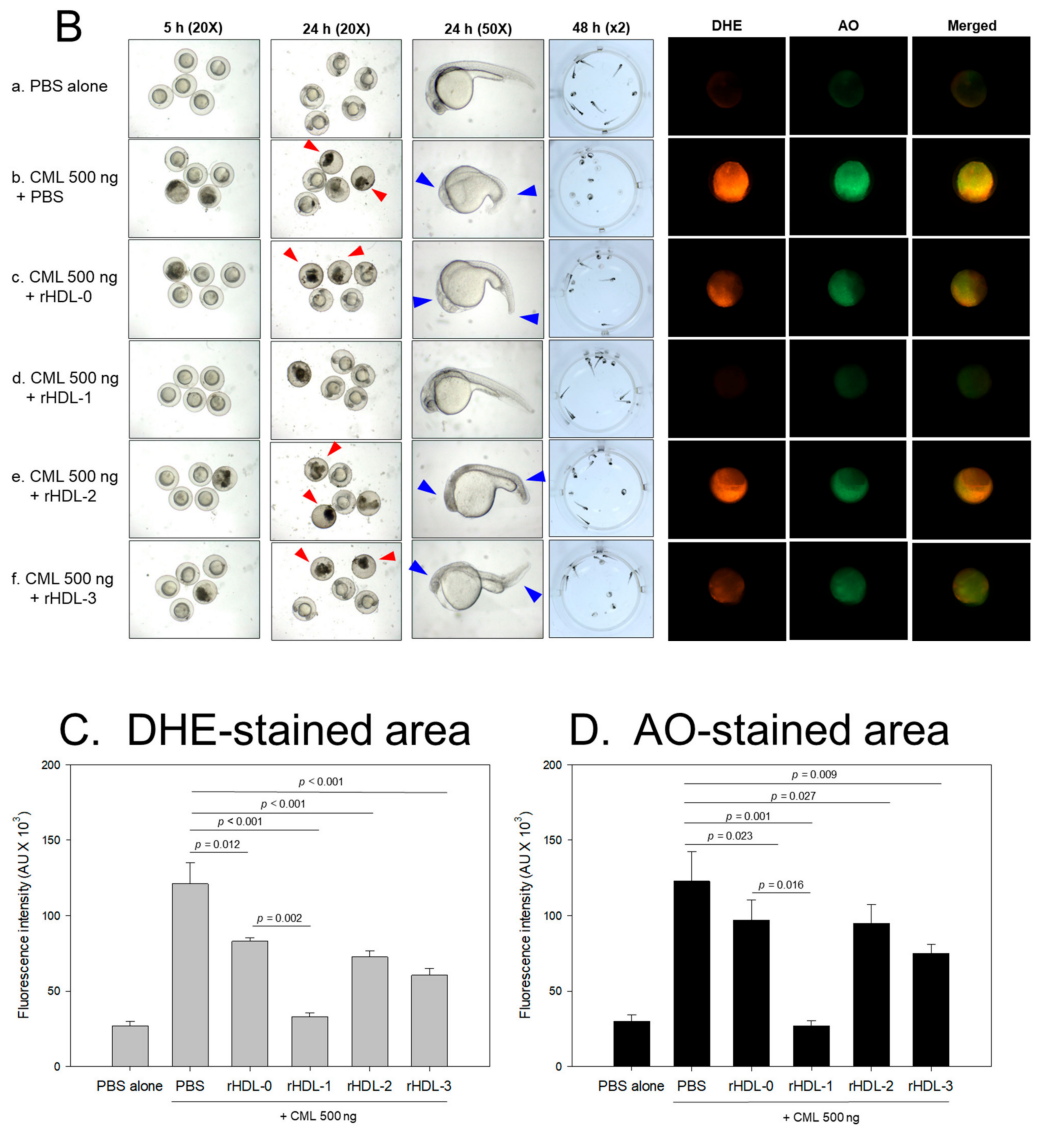
Microinjection of reconstituted high-density lipoproteins (rHDL) containing each policosanol under the presence of carboxymethyllysine (CML) into zebrafish embryo. (**A**) Survivability of zebrafish embryos during 24 h post-injection. * *p* < 0.05 vs. CML + PBS; ** *p* < 0.01 vs. CML + PBS; *** *p* < 0.001 vs. CML + PBS; ^###^ *p* < 0.001 vs. CML + PBS (**B**) Stereoimage observation and fluorescence staining analysis at 5 h and 24 h post-injection. Developmental speed and morphology of embryos were compared by observation of stereo spectroscopy (Zeiss Stemi 305, Oberkochen, Germany). Dihydroethidium (DHE) staining and acridine orange (AO) staining analysis were used to compare ROS production and cellular apoptosis, respectively, at 5 h post-injection. Red arrowhead indicates dead embryo. Blue arrowhead indicates developmental retardation in eye pigmentation and tail elongation. (**C**) Quantification of DHE-stained area (Ex = 585 nm, Em = 615 nm) to compare ROS production in embryos using Image J software (http://rsb.info.nih.gov/ij/ accessed on 8 June 2023). AU, arbitrary units. (**D**) Quantification of the AO-stained area (Ex = 505 nm, Em = 535 nm) to compare the apoptosis extent in embryonic cells using Image J software (http://rsb.info.nih.gov/ij/ accessed on 8 June 2023). AU, arbitrary units.

Microinjection of CML (500 ng) into zebrafish embryos exhibited the lowest level of survival (28 ± 2% survivability) at 24 h post-injection (Figure 5A), whereas injection of PBS alone showed the highest survivability of around 87 ± 5%. In the presence of CML, a co-injection of rHDL-1 caused the higher embryo survivability (~81 ± 4%), whereas the co-injection of rHDL-0 resulted in lower survivability of approximately 56 ± 3% (*p* = 0.002). Either rHDL-2 or rHDL-3-injected embryos showed similar survivability (61 ± 5% and 59 ± 4%, respectively) to that of rHDL-0. Even though all rHDL, with or without policosanol, showed adequate protective activity against the CML toxicity, rHDL-1 exerted the most potent activity to recover the highest survivability and fastest development.

The stereoimage of embryos displayed a normal morphology and developmental process in the PBS alone group (Figure 5B). In contrast to this, a severe developmental impairment with stunted growth and deformities of eye pigments and tail elongation was observed in the CML alone group at the 21-somite stage. Co-injection of rHDL-0 with the CML showed a slight improvement in the CML-induced developmental deformities; however, a slow developmental speed and hatching of embryos (~46%) was observed at 48 h post-treatment. Unlike this, the co-administration of rHDL-1 effectively counteracts the developmental abnormalities caused by CML. This is evidenced by the normal appearance of embryo in the primordium-6 stage, typical eye pigmentation, and tail development surpassing 32 somites in all treated embryos, with a 73% hatching ratio at 48 h after post-treatment. The co-injection of rHDL-2 and rHDL-3 had minimal influence on embryo hatching and the recovery of developmental abnormalities caused by the CML. At 24 h post-treatment, there was a noticeable decline in embryo hatching rates, with a 40% decrease in the case of CML co-injected with rHDL-2 and a 60% decrease with rHDL-3, when compared to CML co-injected with rHDL-1. Interestingly, embryos injected with rHDL-2 exhibited the least impact on eye pigmentation (indicated by blue arrows) and tail elongation. The microinjection of CML and rHDL into embryo experiments showed that rHDL-1, which contains PCO1, has a significantly higher impact over PCO2 and PCO3 on the restoration of embryo development and hatching impaired by CML.

Dihydroethidium (DHE) staining and acridine orange (AO) staining revealed that the CML + PBS group (photograph b) showed the strongest red and green intensity, respectively, suggesting that the injection of CML induced the highest level of reactive oxygen species (ROS) produced and apoptosis extent (Figure 5B). On the other hand, the CML + rHDL-1 group showed the weakest red and green intensity, suggesting that the co-presence of Cuban policosanol in rHDL inhibited the ROS production and attenuated cellular apoptosis.

Dihydroethidium (DHE) staining to detect ROS showed that the CML injection caused a 4.6-fold larger increase in ROS production than the PBS alone group (Figure 5C). Although the co-injection of rHDL-0 resulted in adequate activity to inhibit ROS production by approximately 32%, a co-injection of rHDL-1 caused the lowest ROS production (~74% more reduction of ROS than the CML alone group). The co-injection of rHDL-2 and rHDL-3 groups showed a similar extent of ROS production with rHDL-0 (~41–49% more reduction than the CML alone group). As shown in Figure 5D, acridine orange (AO) staining to detect cellular apoptosis showed that the CML alone group had a 4.2-fold larger increase in the apoptosis extent than the PBS group, indicating that acute cell death occurred by a CML injection. On the other hand, co-injection of rHDL-1 resulted in the least apoptosis (~80% more reduction of apoptosis than the CML alone group), while a co-injection of the rHDL-0 group showed a 20% reduction. Interestingly, the rHDL-2 and rHDL-3 groups showed a similar extent of apoptosis with that of the rHDL-0 group (~23% and 39% lower than the CML alone group). This result indicates that the cytoprotective effect of rHDL was enhanced by incorporating policosanol, particularly PCO1 (Cuban policosanol, Raydel^®^). These results suggest that the anti-oxidant and anti-apoptosis activity of rHDL alone could be enhanced by incorporating PCO1 into the core of HDL, indicating that the quality of rHDL was affected by the type of policosanol depending on the sources and origins.

### 2.7. Recovery from Acute Paralysis of CML Toxicity

**Figure 6:**
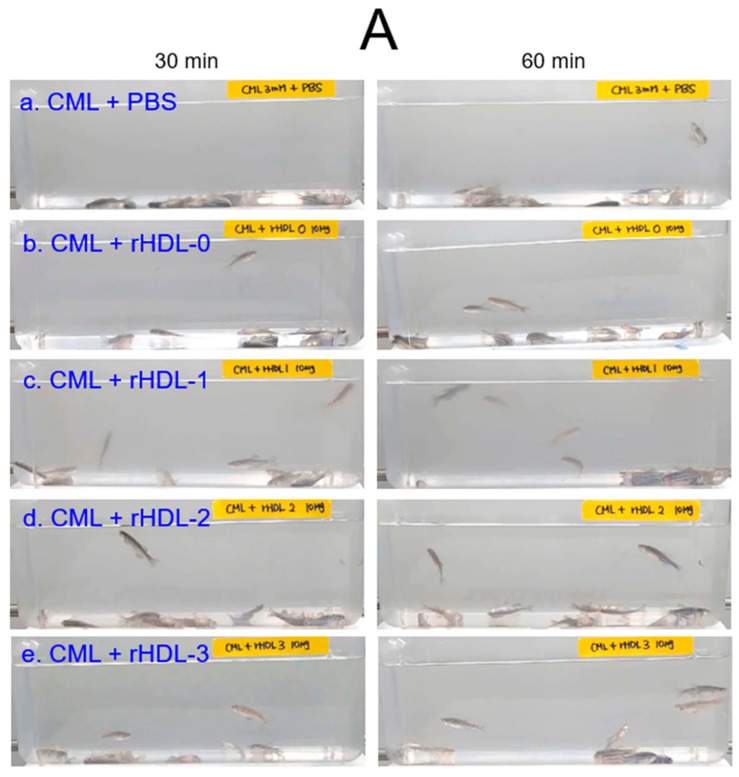

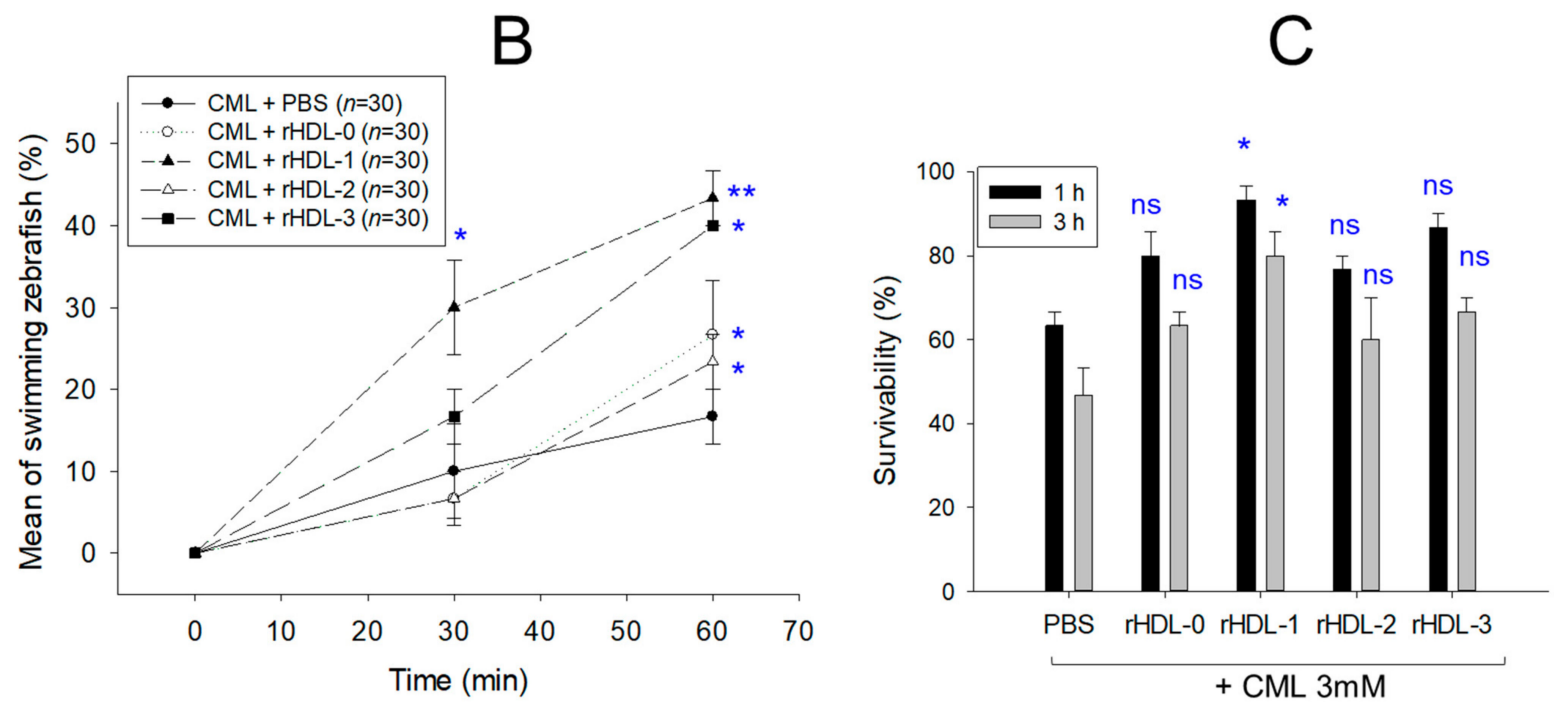
Observation of the swimming ability and survivability after an injection of carboxymethyllysine (CML) with or without reconstituted high-density lipoproteins (rHDL). (**A**) Still image of the swimming pattern of zebrafish after 30-min and 60-min post-injection of CML (250 μg) and each rHDL (10 μg of protein) per fish. (**B**) Percentage of swimming zebrafish after 30 and 60 min post-injection of CML (250 μg) and each rHDL. (**C**) Survivability at 1 h and 3 h post-injection of CML (500 μg) and each rHDL (10 μg). * *p* < 0.05 vs. CML + PBS; ** *p* < 0.01 vs. CML + PBS; ns, not significant.

An intraperitoneal (IP) injection of carboxymethyllysine (CML, 250 μg, final 3 mM in zebrafish body weight around 300 mg) into adult zebrafish (~12 weeks old) caused acute paralysis at 30 min post-injection. All zebrafish in the CML alone group (photograph a) could not swim and were lying down on the bottom of the tank with occasional quivering (Figure 6A), in spite of they were still alive but trembling at 30 min post-injection. At 60 min, 16% of fish could swim again, with 60% survivability in the CML alone group, but the swimming pattern involved wobbling, seizure, and uncontrollable movements (Supplementary Video S1). On the other hand, the rHDL-1 co-injected group showed the fastest recovery of swimming ability; ~43% of fish could swim again, displaying a more active and natural swimming pattern and 90% survivability at 60 min post-injection (Supplementary Video S2). The first death of the was fish detected at 6 ± 3 min and 28 ± 5 min post-injection in the CML + PBS group and CML + rHDL-1 group, respectively, suggesting that the rHDL-1 treatment induced surprisingly attenuated paralysis of neurotoxicity and protected the zebrafish from acute death caused by the CML injection.

The other rHDL group showed a similar extent of recovery for swimming ability; approximately 6–7% of fish could swim again in the rHDL-0 and rHDL-2 group at 30 min post-injection (Figure 6B), while 16 ± 3% of fish recovered their swimming ability in the rHDL-3 group. At 60 min post-injection, the CML alone group showed that 16 ± 3% of fish exhibited swimming ability. In contrast, although the CML + rHDL-1 group showed the highest recovery of swimming ability, around 43 ± 3% of the fish could swim again. Under CML, the rHDL-0 group and the rHDL-2 group showed similar recovery extent of swimming ability around 23–26%, while the rHDL-3 group showed that 40 ± 1% of fish could swim again.

As shown in Figure 6C, all groups showed a decrease in survivability in a time-dependent manner after the IP injection of CML. At one hr post-injection, the CML + PBS group showed the lowest survivability of approximately 63 ± 3%, while a co-injection rHDL increased the survivability to approximately 76–93%. In particular, the CML + rHDL-1 group showed up to 93 ± 3% survivability, suggesting that Raydel policosanol exerted the strongest anti-inflammatory activity to neutralize CML toxicity. At 3 h post-injection, the CML + PBS group showed the lowest survivability of approximately 46 ± 6%, while the CML + rHDL-1 group showed the highest survivability (~80 ± 5%). Interestingly, the rHDL-2 and rHDL-3 groups showed 60 ± 10% and 66 ± 3% survivability, respectively, which were similar to that of the rHDL-0 group of approximately 63 ± 3% survivability.

### 2.8. Histology Analysis of Hepatic Tissue with H&E Staining

**Figure 7:**
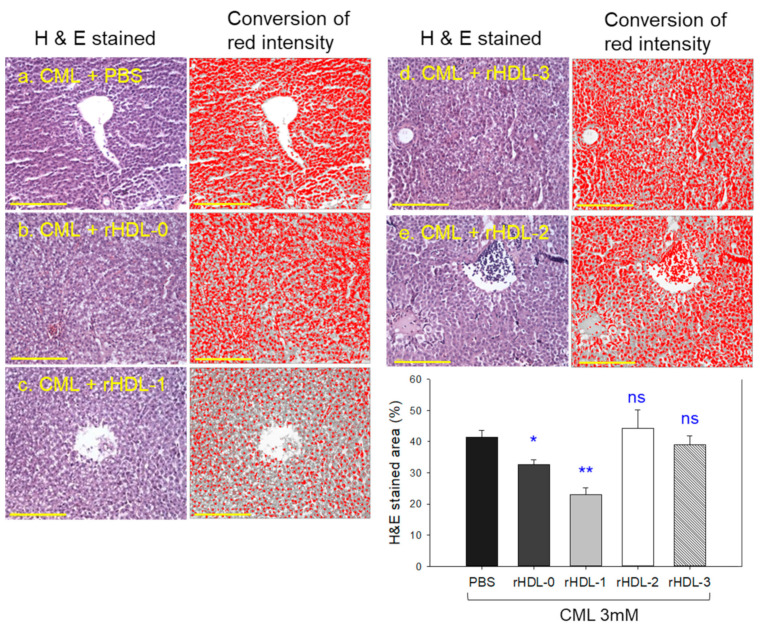
Histology analysis of hepatic tissue from zebrafish injected with carboxymethyllysine (CML) and each reconstituted high-density lipoprotein (rHDL). Photographs a to e show an image of the infiltration of neutrophils by hematoxylin and eosin (H&E) staining and conversion of red intensity. The yellow scale bar indicates 100 μm. The inset graph shows quantification of the nucleus area from the H&E staining using Image J software (http://rsb.info.nih.gov/ij/ accessed on 16 May 2023). * *p* < 0.05 vs. PBS; ** *p* < 0.01 vs. PBS; ns, not significant.

Histology analysis with Hematoxylin & Eosin (H&E) staining revealed the CML alone group (CML + PBS, photograph a) to have the most intense red stained area (Figure 7a), approximately 42 ± 3% of red intensity, indicating the highest number of infiltrated neutrophils. On the other hand, the CML + rHDL-0 group (photograph b) showed a significantly reduced stained area of approximately 32 ± 2% red intensity, suggesting that rHDL could exert adequate anti-inflammatory activity of up to 24% less stained area than the CML + PBS group (*p* = 0.032). In contrast, the CML + rHDL-1 group (photograph c) showed the lowest number of neutrophils (~23 ± 2% red intensity), which was 46% less stained area than the CML + PBS group (*p* = 0.006). Interestingly, the CML + rHDL-2 (photograph d) and CML + rHDL-3 (photograph e) groups did not exhibit anti-inflammatory activity. They showed a similar H&E-stained pattern to the CML + PBS group: approximately 44% and 39% of stained area, respectively. These results suggest that incorporating PCO1 enhanced the anti-inflammatory activity in rHDL, while PCO_2_ and PCO_3_ did not.

### 2.9. Extent of Fatty Liver Changes, ROS Production, and Apoptosis in Hepatic Tissue

**Figure 8:**
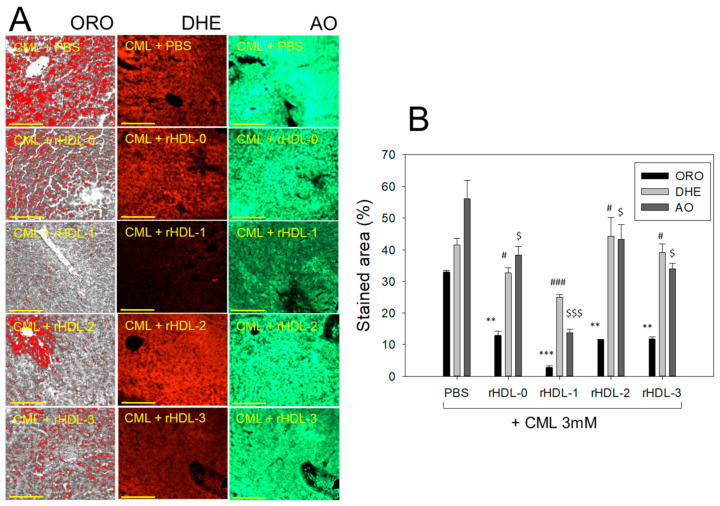
Comparative effect of reconstituted high-density lipoproteins (rHDL) comprising policosanol against carboxymethyllysine (CML) altered fatty liver changes, reactive oxygen species (ROS) generation, and apoptosis in the liver of adult zebrafish. (**A**) Histological images of oil red O staining (ORO), dihydroethidium (DHE) staining, and acridine orange (AO) staining representing fatty liver changes, ROS generation, and extent of apoptosis, respectively. DHE fluorescent images were captured at an excitation wavelength of 585 nm and emission wavelength of 615 nm, while AO fluorescent-stained images were visualized at excitation and emission wavelength of 505 nm and 535 nm. The yellow scale bar indicates 100 μm. (**B**) Quantification of the stained area by employing Image J software (http://rsb.info.nih.gov/ij/accessed on 16 May 2023). *t*-test was employed to establish the statistical difference between the groups with respect to CML + PBS (control) group. *** *p* < 0.001, ** *p* < 0.01 for ORO staining, while ^#^ *p* < 0.05, ^###^ *p* < 0.001 for DHE staining and ^$^ *p* < 0.05, ^$$$^ *p* < 0.001 for AO staining.

As shown in Figure 8A, Oil red O staining showed that the CML + PBS group exhibited the highest red stained area, approximately 2.5-fold higher than the CML + rHDL-0 group. The CML + rHDL-1 group showed the weakest red intensity, 92% lower than the CML + PBS group. In contrast, the other rHDL group showed 65% lower red intensity than the CML + PBS group (Figure 8B). This result suggests that rHDL-1 exerted the strongest inhibition ability to prevent fatty liver changes, even though all rHDL showed adequate inhibition activity. DHE staining also showed that the CML + PBS group showed the highest red fluorescence to represent approximately 42 ± 2% ROS production, while the CML + rHDL-1 group showed the lowest ROS production (~43% lower (*p* < 0.001) than the CML + PBS group). Other rHDL groups showed 22–25% lower ROS production (*p* < 0.05) than the CML + PBS group, indicating that all rHDL exerted considerable inhibition against ROS production in the presence of CML. AO staining also showed that the CML + PBS group had the highest green fluorescence area to represent the extent of cellular apoptosis, approximately 56 ± 5%. In contrast, the CML + rHDL-1 group showed the lowest ROS production (~77% lower than the CML + PBS group). The other rHDL group showed 24–42% lower ROS production than the CML + PBS group, suggesting that rHDL could protect hepatic cells from apoptosis. Overall, Raydel policosanol in rHDL-1 showed the highest protection of hepatic tissue from fatty liver changes, ROS production, and cellular apoptosis, whereas the other policosanols in rHDL-2 and rHDL-3 showed much less protection activity, similar to rHDL-0.

### 2.10. Immunohistochemistry with IL-6 Analysis of Hepatic Tissue

**Figure 9:**
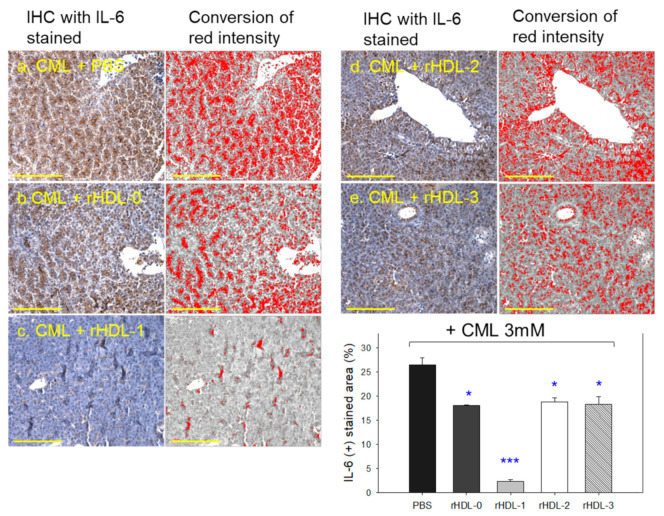
Microscopic observations of the IL-6 stained area from immunohistochemistry (IHC) among each rHDL injected zebrafish. Photographs a to e show representative images of IHC using IL-6 antibody-stained hepatic tissue. The yellow scale bar indicates 100 μm. The inset graph quantifies the IL-6 antibody-stained area with brown intensity using Image J software (http://rsb.info.nih.gov/ij/ accessed on 20 May 2023). IL-6, interleukin-6; rHDL, reconstituted high-density lipoproteins. * *p* < 0.05 vs. PBS; *** *p* < 0.001 vs. PBS.

As shown in photos of Figure 9, immunohistochemical detection of interleukin (IL)-6 with hepatic tissue revealed the CML alone group to exhibit the largest stained area (~26.4%), while the CML + rHDL-1 group showed the smallest IL-6-stained area of only 2.4%. Interestingly, the rHDL-0, rHDL-2, and rHDL-3 groups showed a similar level of IL-6-stained area ~18–19%, which was 29% lower (*p* < 0.05) than that of the CML + PBS group (inset graph of Figure 9).

CML + rHDL-1 group showed 11-fold lower (*p* < 0.001) IL-6-stained area than the CML + PBS group, indicating that the Cuban policosanol showed the strongest anti-inflammatory activity. Other policosanols in rHDL-2 and rHDL-3 showed much less anti-inflammatory activity that was similar to rHDL-0, suggesting that the incorporation of Chinese policosanol and American policosanol in rHDL did not improve the anti-inflammatory activity.

### 2.11. Change in the Lipid Profile after an IP Injection of CML and Each rHDL

**Figure 10:**
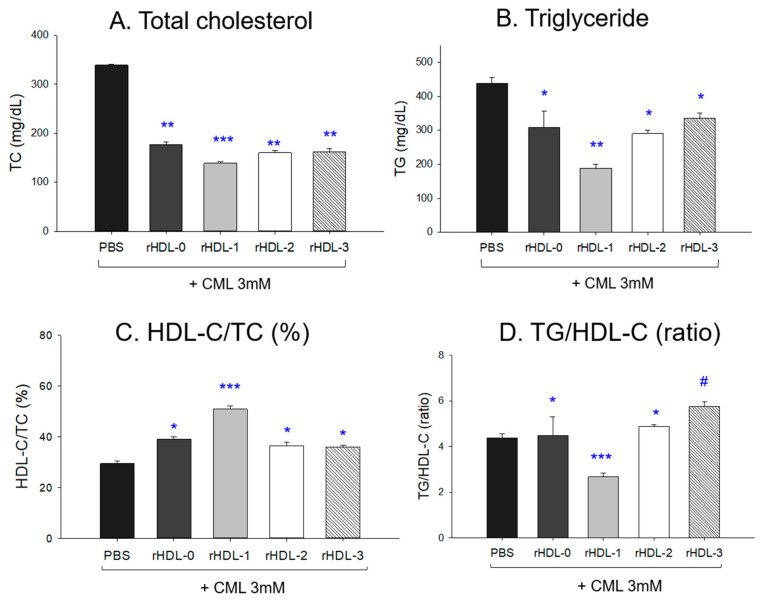
Quantification of lipid profiles from zebrafish plasma. All zebrafish were sacrificed at 180 min post injections of CML and each rHDL. * *p* < 0.05 vs. PBS; ** *p* < 0.01 vs. PBS; *** *p* < 0.001 vs. PBS; # *p* < 0.05 vs. PBS. CML, carboxymethyllysine; TC, total cholesterol; TG, triglyceride; HDL-C, high-density lipoproteins; rHDL, reconstituted high-density lipoproteins.

After collecting plasma from each zebrafish group, quantifying plasma lipid revealed that the CML + PBS group had the highest plasma total cholesterol (TC) and triglyceride (TG) levels, as shown in Figure 10A,B. On the other hand, the CML + rHDL-1 group showed the lowest TC and TG among the groups, 59% and 57% lower than the CML + PBS group. The other rHDL groups showed adequate lipid-lowering effects, around 48–57% of lower TC and 24–34% lower TG than the CML + PBS group.

The HDL-C/TC (%) was the lowest in the CML + PBS group (~29 ± 1%), while the CML + rHDL-1 group showed the highest HDL-C/TC (%) around 51 ± 2% (Figure 10C). Interestingly, the other rHDL group showed a similar HDL-C/TC (%) level (36–39%). As shown in Figure 9d, in the presence of CML, the rHDL-0, rHDL-2, and rHDL-3 groups showed higher TG/HDL-C levels (approximately 4.48, 4.88, and 5.75, respectively) than the CML + PBS group (~4.39), while the CML + rHDL-1 group showed the lowest ratio (~2.67). These results suggest that Cuban policosanol in rHDL-1 was remarkably effective in raising the HDL-C/TC(%) and lowering the TG/HDL-C ratio. In contrast, Chinese and American policosanol in rHDL-2 and rHDL-3, respectively, were ineffective compared to rHDL-0.

### 2.12. Change of Serum AST and ALT after Injection of CML and Each rHDL

**Figure 11:**
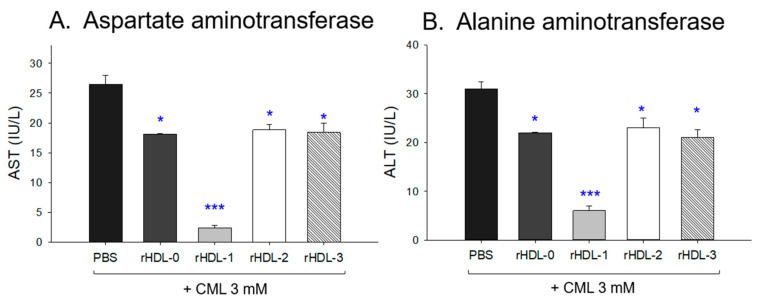
Quantification of hepatic enzymes in zebrafish plasma. All zebrafish were sacrificed at 180 min post injections of CML and each rHDL. * *p* < 0.05 vs. PBS; *** *p* < 0.001 vs. PBS. AST, aspartate aminotransferase; ALT, alanine aminotransferase; CML, carboxymethyllysine; rHDL, reconstituted high-density lipoproteins.

The CML + PBS group showed the highest level of AST and ALT levels around 26–31 IU/L (Figure 11), while the CML + rHDL-1 group showed the lowest levels of AST and ALT around 3–6 IU/L. Other rHDLs showed similar levels of AST and ALT around 18–23 IU/L, regardless of whether they contain policosanol. These results showed that a co-injection of rHDL containing Raydel policosanol ameliorated the hepatic damage by the CML injection, whereas other policosanols were ineffective, showing a similar extent of amelioration to that of rHDL alone.

## 3. Discussion

Optimally higher HDL-C, >70 mg/dL is associated with a lower mortality: Copenhagen General Population Study (*n* = 116,508) showed that the all-cause mortality was lowest in 73 and 93 mg/dL for men and women, respectively [24]. On the contrary, however, extraordinarily high HDL-C levels, >80 mg/dL, were correlated with more elevated risk of all-cause death and cardiovascular mortality among men but not in women in the general population who do not have coronary artery disease [25]. These results strongly suggest that just increasing the serum level of HDL-C quantity might not help to achieve healthy longevity. Moreover, an enhanced HDL quality and functionality should be accompanied by an elevated HDL-C quantity to ensure healthy longevity.

Because policosanol showed extremely low solubility in water or aqueous buffer, the antioxidant activity of policosanol alone has not been reported successfully so far. Policosanol was incorporated into reconstituted HDL in the current study to overcome this hurdle. Policosanol and its ingredients, long-chain aliphatic alcohols, should not interfere with the native apoA-I structure and normal HDL functionality, such as particle formation after uptake from the intestinal mucosal barrier by binding with apoA-I, antioxidant ability, anti-glycation activity, and anti-inflammatory activity [8,9,17]. Although the reason for the beige color in PCO1 powder (Table 1) has not been explained exactly yet, it has been postulated that the unique color (beige) might be originated from more double bonds, and the higher unsaturated extent of minor components in fatty acids, such as aldehydes and fatty alcohols in PCO1.

The binding with apoA-I, HDL particle shape, size, electromobility, and functionality of the three policosanols after incorporation into rHDL were compared. An rHDL containing PCO1 (Cuban policosanol, Raydel^®^) showed the strongest apoA-I band intensity and the highest blue-shifted WMF (Table 2). The other policosanol showed weaker apoA-I band intensity and smaller particle size of rHDL (Figure 1; Figure 2). These results suggest that Cuban policosanol exhibits the highest binding affinity with the amphipathic domain of apoA-I to form more stabilized and larger HDL particles. The stabilized rHDL-1 exerted the highest activity to prevent HDL_3_ degradation from glycation (Figure 3) and LDL degradation from oxidation (Figure 4). The rHDL-1 group (containing PCO1, Raydel policosanol) showed potent anti-inflammatory activity with higher survivability and less ROS production in zebrafish embryos and adults against CML-mediated hyperinflammation (Figure 5 and Figure 6).

The current results clearly showed that Cuban policosanol has significantly different ingredient compositions and physiological activities to enhance HDL quality and HDL functionalities from in vitro and in vivo experiments. In contrast, the other policosanols did not improve the rHDL regarding HDL particle quality and functionality. Although it is difficult to find what differences in the compositions among policosanols affect the abilities of rHDL in vitro and in vivo, some of the specific ratios of ingredients are distinctly different among the policosanols. Gas chromatography (Table 1) showed that Cuban policosanol (PCO1) had the highest total amount of aliphatic alcohols and 1-octacosanol content (692 mg/g, ~70% in total amount), while Chinese policosanol (PCO2) had the lowest octacosanol content of 56 mg/g (~7.6%). These dramatic differences might affect the binding with apoA-I and particle formation ability in rHDL (lane 2, Figure 1).

Results of the antioxidant activity and anti-glycation activity of rHDL-1 followed the previous reports [8], where rHDL comprising Cuban policosanol (final 10 μM) prevented LDL oxidation. The policosanol comprising rHDL showed higher anti-glycation efficacy in contrast to Chinese policosanol, irrespective of their origin source (sugar cane or rice bran) [17]. Consistent with this, consumption of Cuban policosanol (10 mg/day) efficiently prevented glycation of HDL in middle-aged individuals from randomized, double-blinded, and placebo-controlled study [26]. Also, substantial policosanol consumption (10–20 mg/day) for up to 24 weeks displayed inhibition of LDL oxidation and HDL glycation in healthy Korean individuals with prehypertension.

Moreover, 12 weeks of Raydel policosanol consumption (20 mg/day) in middle-aged healthy Japanese participants revealed a significant reduction of oxidation in the VLDL and LDL and glycated hemoglobin (Hb_A1c_) levels with significant elevation of HDL-associated antioxidant abilities, such as paraoxonase and ferric ion reduction ability [9]. The protection of the liver function by policosanol consumption in a human clinical study was associated well with a decrease in neutrophil infiltration, fatty liver change, and ROS production in hyperlipidemic zebrafish that had consumed Cuban policosanol for eight weeks. These increases in HDL-C quantity and HDL functionality results showed good agreement with previous in vitro studies [8,17], animal experiments [27], and human clinical studies [9,26]; the in vitro potential of policosanol could be enhanced by incorporation into rHDL. The in vitro potentials have been linked with the in vivo efficacy in animal and human clinical studies to lower the blood pressure and protect the liver function via the anti-glycation and anti-oxidant activities of HDL.

An IP injection of CML into hyperlipidemic zebrafish caused severe inflammatory damage in hepatic tissue: more neutrophil infiltration (Figure 7), more fatty liver changes and ROS production (Figure 8), and higher IL-6 expression (Figure 9), as reported elsewhere [23]. Interestingly, the current study found that acute hyperinflammation by an IP injection of CML could be reduced by rHDL, especially that containing PCO1 (Raydel policosanol), showing a nine-fold lower level of IL-6 production than those of PCO2 (Shaanxi policosanol) and PCO3 (Garuda policosanol). The raised IL-6 level is often associated with dyslipidemia, evidenced by reduced HDL-C and increased TG levels in individuals with the inflammatory disease [28,29]. Similarly, an elevated IL-6 level, and associated enhancement of TG and LDL-C levels were observed in individuals with psoriatic arthritis than psoriasis alone [30], signifying IL-6 was associated with dyslipidemia with autoimmune disease patients. The improvement effect of PCO1 in lipid profile as depicted in Figure 10 was associated with potent CETP inhibition activity of PCO1 (Cuban policosanol), as reported previously [8,27]. According to our previous report [27], rHDL containing policosanol showed much stronger CETP inhibition activity: around 4.5-fold higher than policosanol alone in ethanol. Therefore, administering rHDL containing PCO1 could help reduce the acute inflammatory cascade by simultaneously suppressing IL-6, TC, TG, AST, and ALT in the liver and blood.

## 4. Materials and Methods

### 4.1. Materials

Palmitoyloleoyl phosphatidylcholine (POPC, #850457) was acquired from Avanti Polar Lipids (Alabaster, AL, USA). Fructose (CAS-No 57-48-7, Cat #F0127), *N*-ε-carboxymethyllysine (CAS-No 941689-36-7, Cat#14580-5g), sodium cholate (#C1254), CuSO_4_ (Sigma # 451657), malondialdehyde (Sigma # 63287), and 2-phenoxyethanol (Sigma P1126) were procured from Sigma–Aldrich (St. Louis, MO, USA). The Cuban policosanol (PCO_1_), from sugar cane wax alcohol, was obtained from the National Center for Scientific Research (CNIC, Havana, Cuba) via Raydel Pty, Ltd. (Thornleigh, NSW, Australia). Chinese policosanol (PCO_2_) from rice bran and American policosanol (PCO_3_) from sugar cane wax were procured from Shaanxi Pioneer Biotech (Xi’an, China) and Garuda International (Exeter, CA, USA). All raw materials of each policosanol were analyzed by gas chromatography as described earlier [17].

### 4.2. Purification of Lipoproteins

The different serum lipoproteins fractions, i.e., LDL (1.019 < d < 1.063), and HDL (1.063 < d < 1.225) were isolated from the blood samples of healthy individuals (average age 23 ± 2 year). The blood was voluntarily donated by the individuals after 12 h fasting and collected according to Helsinki guidelines approved by the Institutional Review Board of Yeungnam University (sanction code 7002016-A-2016-021, sanction date 4 July 2016). Different fractions of lipoproteins from the blood were segregated by density gradient ultracentrifugation, where different density zones were prepared by using NaCl and NaBr following the standard method [31]. In brief, serum (plasma) was ultracentrifuged at 100,000× *g* for 24 h at 10 °C. The separated lipoproteins were individually collected and processed for dialysis to remove traces of NaBr. Dialysis was performed for 24 h at constant agitating conditions using Tris-buffered saline (pH 8.0).

### 4.3. Purification of Human apoA-I

According to the earlier described method [32], the lipid-free apoA-I fraction was isolated from HDL_2_ using ultracentrifugation, column chromatography, and liquid-liquid extraction techniques. Finally, SDS-PAGE was employed to examine the purity of isolated apoA-I (>95% purity).

### 4.4. Reconstituted HDL Synthesis

Reconstituted HDL (rHDL) was synthesized by blending POPC, cholesterol, apoA-I, and policosanol at 95:5:1:0 or 95:5:1:1 molar ratio following sodium cholate dialysis procedure [17]. Finally, residual endotoxin (3.1–3.3 EU/mL) was quantified utilizing a commercial endotoxin quantification kit (BioWhittaker, Walkersville, MD, USA) following the standard method suggested by the supplier.

### 4.5. Protein Quantifiaction

The protein concentrations of the isolated LDL, HDL, and reconstituted HDL (rHDL) were quantified by the Lowry method as modified by Markwell et al. [33], while the Bradford method (Quick Start™ Bradford Protein Assay Kit, Bio-Rad #5000201) was employed for protein quantification in lipid-free apoA-I. Bovine serum albumin (BSA) was used as a reference for the protein quantification in both assays. 

### 4.6. Electromobility Analysis in Agarose

The electrophoretic mobility of various combinations of rHDL was accessed by agarose gel electrophoresis employing non-denaturing conditions. rHDL was loaded in 0.6% agarose gel, and electrophoresis was carried out for 1 h at 50 V using 1 × TAE buffer. Finally, the gel was stained with Coomassie brilliant blue (final 1.25%) to visualize the separated bands.

### 4.7. Tryptophan Fluorescence Characterization in the rHDL

The wavelengths of maximum fluorescence (WMF) of the intrinsic tryptophan in apoA-I in the lipid-free and the lipid-bound state were measured by using a spectrofluorometer (Perkin-Elmer, Norwalk, CT, USA) equipped with Spectrum FL software version 1.2.0.583 (Perkin-Elmer). In brief, a protein solution in a 1 cm pathlength quartz cuvette exited at 295 nm to avoid Tyrosine fluorescence, and the emission fluorescence spectra were scanned at 310 to 360 nm.

### 4.8. LDL Oxidation Assay

The effect of rHDL to prevent CuSO_4_-induced LDL oxidation (oxLDL) was examined by agarose gel electrophoresis and quantified by thiobarbituric acid reactive substances (TBARS) assay. Human LDL (8 μg of protein) was mixed with CuSO_4_ (10 μM) and, after that, treated with rHDL (0.5 μg of protein). The mixture was incubated at 37 °C for 4 h, followed by 0.22 μm syringe filtration. The filtered content was processed for TBARS assay to examine the degree of oxidation using malondialdehyde (MDA) as a reference following the earlier described method [34].

The oxidized LDL with CuSO_4_ and subsequently treated with rHDL was also assessed in agarose gel electrophoresis to examine the oxidative damage following the earlier described method [35]. In brief, each sample was loaded in 0.5% agarose gel under non-denatured conditions and electrophoresed at 50 V for 1 h using Tris-acetate-EDTA buffer (pH 8.0). Finally, the gel was stained with Coomassie brilliant blue (final 1.25%) to visualize the apo-B fragment in the LDL. The faster electrophoretic mobility suggests the oxidative damage of LDL owing to structural changes that impact the charge per unit length of the apo-B degradation.

### 4.9. Electron Microscopic Examination

The particle diameter of different rHDL was examined using transmission electron microscopy (TEM, Hitachi, model HT-7800; Ibaraki, Japan) after negative staining with sodium phosphotungstate (PTA). In brief, 5 μL of rHDL (0.3 mg/mL final) was mixed with 5 μL of 1% PTA (pH 7.4). A 5 μL of the sample mixture was embedded over a 300-mesh copper grid and air-dried, the excess content was blotted, and the morphology was visualized under TEM at 40,000× magnification at 80 kV acceleration.

### 4.10. Glycation of HDL in the Presence of rHDL

The HDL (2 mg/mL) was mixed with fructose (final 250 mM) in potassium phosphate/0.02% sodium azide buffer (pH 7.4) in the presence and absence of rHDL following the previously described method [17]. The mixture was incubated for 4 h at 37 °C in 5% atmospheric CO_2_. The degree of advanced glycation reactions was evaluated by monitoring the fluorescent intensity using a spectrofluorometer (Perkin-Elmer, Norwalk, CT, USA) at the excitation wavelength of 370 nm and emission wavelength of 440 nm following the previously described method [36].

### 4.11. Maintenance of Zebrafish

The normative protocol [37] for the Care and Use of Laboratory Animals [38] endorsed by the Committee of Animal Care and Use of Raydel Research Institute (approval code RRI-20-003, Daegu, Korea) was utilized to maintain zebrafish and embryos. A normal terabit flake (TetrabitGmbh D49304, Melle, Germany) was used to feed the zebrafish that were maintained at dark (10 h) and light (14 h) photoperiods at a constant water temperature (28 °C) in the breeding system.

### 4.12. Microinjection of Zebrafish Embryos

At 4 h post-fertilization (hpf), zebrafish embryos were collected and randomly distributed into six cohorts (130–140 embryos in each group). Group I embryos were administered a 10 μL injection of PBS, while those in Group II received a 500 ng CML/10 μL PBS injection. Embryos in Groups III, IV, V, and VI received 500 ng CML suspended in 10 μL of rHDL-0, rHDL-1, rHDL-2, and rHDL-3, respectively. A stereoimage microscopic (Carl Zeiss Stemi305, Jena, Germany) examination was conducted for all groups, with images captured at 5 h and 24 h post-treatment to evaluate developmental abnormalities.

### 4.13. Acute Inflammation in Adult Zebrafish

Acute inflammation in zebrafish was induced by the injection of 250 μg carboxymethyllysine (CML) in 10 μL PBS (equivalent to 3 mM CML considering zebrafish’s 300 mg average body weight). Zebrafish were randomly divided into five groups (*n* = 30 for each group). Group I zebrafish received 10 μL PBS containing 250 μg CML injection. In comparison, zebrafish in Group II, III, IV, and V were co-injected with 250 μg CML with 10 μL of rHDL-0, rHDL-1, rHDL-2, and rHDL-3, respectively. A 28-gauge needle was used for injection, which was inserted into the abdominal region of zebrafish after anesthetizing them into 0.1% of 2-phenoxyethanol. Zebrafish swimming and survivability were analyzed at 30 min and 60 min post-treatment as the parameters described earlier [23]. Tail fin motion and death of body convulsion are the primary attribute to evaluate the consequence on swimming activity [39].

### 4.14. Blood Collection and Analysis

For plasma lipid analysis, blood was accumulated from zebrafish of distinct groups. In brief, 2 μL blood from the zebrafish of various groups was collected and instantly mingled with 3 μL PBS comprising ethylenediaminetetraacetic acid (EDTA, final 1 mM), followed by 15 min centrifugation at 5000× *g* and the supernatant managed for the quantification of total cholesterol (TC) and triglyceride (TG) employing a colorimetric assay kit (Cholesterol, T-CHO and TG, Cleantech TS-S; Wako Pure Chemical, Osaka, Japan). HDL-C (AM-202), alanine transaminase (ALT) (AM-103K), and aspartate transaminase (AST) (AM-201) were estimated using a commercial detection kit (Asan Pharmaceutical, Hwasung, Korea), succeeding the mentioned methodology stipulated by the manufacturer.

### 4.15. Histological Examination

The liver of the zebrafish from distinct groups was extracted surgically after sacrificing them. The liver tissue was conserved in 10% formalin for 24 h succeeding ethanol dehydration. The dehydrated tissue was inserted in paraffin, succeeded by 5 μm thick sectioning that was successively treated with poly-L-lysine and smeared with Hematoxylin and Eosin (H&E). To inspect morphological changes, the stained tissue was observed under an optical microscope (Motic Microscopy PA53MET, Hong Kong, China). Image J platform (http://rsb.info.nih.gov/ij/ accessed on 15 September 2022) was employed to compute the nucleus-stained area by transforming the native H&E-stained nucleus to red intensity. 

The hepatic tissue was sliced using a microtome (Leica, CM1510s, Heidelberg, Germany) in a 7 μm tissue section and stained for 10 min with oil red O (Cat#O0625, Sigma, St., Louis, MO, USA), pursued by washing with water and ensuing counterstain with hematoxylin. After 2 min, the incubation-stained area was washed with water and envisioned under an optical microscope (Motic Microscopy PA53MET, Hong Kong, China).

Dihydroethidium (DHE) fluorescent staining was implemented to compute the ROS generation [40]. The 5 μm thick tissue section was stained with DHE (cat # 37291; Sigma, St. Louis, MO, USA) (final 30 μm) for 30 min in the dark. After three times washing with water, the stained section was visualized under a fluorescence microscope (Nikon Eclipse TE2000, Tokyo, Japan), and the fluorescent intensity was computed using the Image J platform (http://rsb.info.nih.gov/ij/ accessed on 15 September 2022). The degree of apoptosis was compared by acridine orange (AO) staining [41]. Briefly, the tissue section was stained with AO and images were captured under fluorescent microscope at excitation wavelength of 505 nm and emission wavelength of 535 nm.

IL-6 production in hepatic tissue was quantified by immunohistochemical staining. Briefly, a 5 μm thick tissue section was detected with primary anti-IL-6 antibody (ab9324, Abcam, London, UK). After overnight incubation at 4 °C, the tissue section was foster using Envision + system kits (code 4001, Dako, Denmark) containing horseradish peroxidase (HRP)-conjugated secondary antibody against the IL-6-specific primary antibody.

### 4.16. Statistical Analysis

Data were presented as mean ± SD from three experiments conducted in duplicated samples. For the zebrafish study, multiple groups were compared using a one-way analysis of variance (ANOVA) between the groups using Dunnett’s test as post hoc analysis. Statistical comparisons amidst the groups were performed using *t* test in SPSS software (version 28.0; SPSS, Inc., Chicago, IL, USA). A *p*-value < 0.05 was considered as significant.

## 5. Conclusions

Among the three policosanols, Cuban policosanol (PCO_1_, Raydel^®^) exhibited the most desirable properties during the in vitro synthesis of rHDL, with the largest particle size and stabilization of apoA-I. The rHDL containing Cuban policosanol exerted remarkably more potent activities than other Chinese policosanol (PCO_2_, Shaanxi) and American policosanol (PCO_3_, Lesstanol^®^): anti-glycation activities against fructation and anti-oxidant abilities to prevent LDL oxidation. The rHDL containing Cuban policosanol also showed the strongest in vivo anti-oxidant and anti-inflammatory activities with the highest survivability in zebrafish embryos and adults by preventing hyperinflammation in the presence of CML. The rHDL containing Cuban policosanol inhibited acute inflammatory cascade via elevation of serum HDL-C and suppression of serum TG, AST, and ALT: the lowest neutrophil infiltration, fatty liver changes, ROS production, and IL-6 levels in hepatic tissue.

## Figures and Tables

**Table 1:** Total wax alcohol contents and ingredient compositions from different products of policosanols.

Product Name/Description	Policosanol 1 (Raydel^®^)		Policosanol 2 (Shaanxi)	Policosanol 3 (Lesstanol^®^)
Country	Cuba		China	USA
Manufacturer	CNIC ^1^		Shaanxi ^2^	Garuda ^3^
Powder image	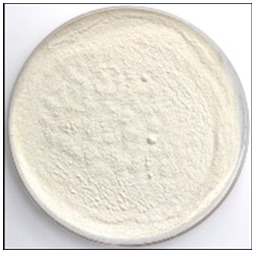		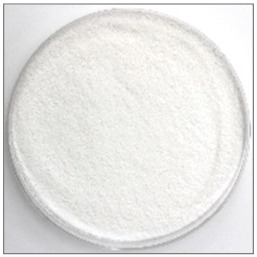	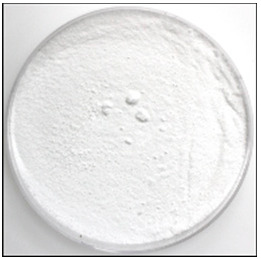
Source	Sugar Cane Wax		Rice Bran	Sugar Cane Wax
Ingredients of Long-Chain Aliphatic Alcohols	Desirable Range ^4^ (mg/g)	Determined Amount (mg/g) (%) ^5^	Determined Amount (mg/g) (%)	Determined Amount (mg/g) (%)
Average molecular weight	418		442	419
Total amount on the label	>900	982	400	900
1-tetracosanol (C24)	0.1–20	0.3 (0.0)	70 (9.5)	28 (3.1)
1-hexacosanol (C26)	30–100	38 (3.9)	58 (7.9)	69 (7.7)
1-heptacosanol (C27)	1–30	9 (0.9)	1 (0.1)	8 (0.9)
1-octacosanol (C28)	600–700	692 (70.5)	56 (7.6)	546 (60.7)
1-nonacosanol (C29)	1–20	6 (0.6)	6 (0.8)	11 (1.2)
1-triacotanol (C30)	100–150	139 (14.2)	213 (28.8)	131 (14.6)
1-dotriacotanol (C32)	50–100	78 (7.9)	181 (24.5)	62 (6.9)
1-tetratriacotanol (C34)	1–50	20 (2.0)	154 (20.8)	45 (5.0)
Determined final total amount (mg)	more than 900	982 (100)	739 (100)	900 (100)

^1^ CNIC, National Center for Scientific Research (CNIC), Habana, Cuba. ^2^ Shaanxi, Shaanxi Pioneer Biotech, Xi’an, China. ^3^ Garuda, Garuda International, Exeter, CA, USA. ^4^ adopted from a previous paper [15]. ^5^ Percentages (in parentheses) of the determined amount.

**Table 2:** Characterization of rHDL containing policosanol from different sources.

Name	Description	MW of PCO (Averaged)	Molar Ratio POPC:FC:apoA-I:PCO	WMF (nm)	Diameter (nm)
rHDL-0	rHDL alone	-	95:5:1:0	329.2 ± 1.7	60.7 ± 1.5
rHDL-1	Policosanol 1-rHDL	418.0	95:5:1:1	325.2 ± 1.8 ***	83.1 ± 3.1 ***
rHDL-2	Policosanol 2-rHDL	442.4	95:5:1:1	326.5 ± 1.5 *	62.7 ± 2.6 *
rHDL-3	Policosanol 3-rHDL	418.8	95:5:1:1	327.3 ± 1.4 *	60.2 ± 1.6 *

PCO, policosanol; MW, molecular weight (averaged); POPC, palmitoyloleoyl phosphatidylcholine; FC, free cholesterol; rHDL, reconstituted high-density lipoproteins; WMF, wavelength maximum fluorescence (Ex = 295 nm, Em = 305–400 nm). Policosanol 1, Cuban policosanol (Raydel^®^); Policosanol 2, Chinese policosanol (Shaanxi); Policosanol 3, American policosanol (Garuda International, Lesstanol^®^). *** *p* < 0.001 vs. rHDL-0; * *p* < 0.05 vs. rHDL-0.

## Data Availability

The data used to support the findings of this study are available from the corresponding author upon reasonable request.

## Supplementary Materials

The following supporting information can be downloaded at: https://www.mdpi.com/article/10.3390/molecules28186715/s1. Video S1. Paralyzed moving pattern of carboxymethyllysine (CML) alone-injected zebrafish. Video S2. Active swimming pattern of CML + rHDL-1 injected zebrafish.
